# Bioactive Glasses as Carriers of Cancer-Targeted Drugs: Challenges and Opportunities in Bone Cancer Treatment

**DOI:** 10.3390/ma15249082

**Published:** 2022-12-19

**Authors:** Roger Borges, Agatha Maria Pelosine, Ana Carolina Santos de Souza, Joel Machado, Giselle Zenker Justo, Lionel Fernel Gamarra, Juliana Marchi

**Affiliations:** 1 Centro de Ciências Naturais e Humanas, Universidade Federal do ABC, Santo André 09210-580, Brazil; 2 Departamento de Ciências Biológicas, Universidade Federal de São Paulo, Diadema 05508-070, Brazil; 3 Departamento de Bioquímica, Universidade Federal de São Paulo, São Paulo 05508-070, Brazil; 4 Hospital Israelita Albert Einstein, São Paulo 05652-900, Brazil

**Keywords:** bioactive glasses, bone cancer, molecular-targeted therapy, bisphosphonates, drug delivery

## Abstract

The treatment of bone cancer involves tumor resection followed by bone reconstruction of the defect caused by the tumor using biomaterials. Additionally, post-surgery protocols cover chemotherapy, radiotherapy, or drug administration, which are employed as adjuvant treatments to prevent tumor recurrence. In this work, we reviewed new strategies for bone cancer treatment based on bioactive glasses as carriers of cancer-targeted and other drugs that are intended for bone regeneration in conjunction with adjuvant treatments. Drugs used in combination with bioactive glasses can be classified into cancer-target, osteoclast-target, and new therapies (such as gene delivery and bioinorganic). Microparticulated, nanoparticulated, or mesoporous bioactive glasses have been used as drug-delivery systems. Additionally, surface modification through functionalization or the production of composites based on polymers and hydrogels has been employed to improve drug-release kinetics. Overall, although different drugs and drug delivery systems have been developed, there is still room for new studies involving kinase inhibitors or antibody-conjugated drugs, as these drugs have been poorly explored in combination with bioactive glasses.

## 1. Introduction

Cancer is a complex disease resulting from a series of genetic and epigenetic alterations that lead to a series of changes in the physiology of healthy cells and tissues, an unbalanced tumor-suppressive microenvironment, and excessive cell growth. Among the several types of cancer, bone cancer is typically rare and very debilitating to the patient. Furthermore, most bone malignancies are caused by metastasis from other tissues and organs, which are responsible for 99% of reported cases. Since bone is a highly vascularized tissue because of hematopoiesis (the formation of blood cells), it becomes susceptible to the metastasis of cancers that have a higher incidence, such as breast, prostate, and pancreatic tumors [1].

One of the main characteristics of bone cancer development is the establishment of a vicious cycle involving a molecular and signaling relationship between osteoclasts, osteoblasts, and the cancer cells in the bone microenvironment. In healthy tissue, bone homeostasis happens through the balanced action of bone deposition by osteoblasts and bone resorption by osteoclasts, which is mainly regulated by the receptor activator of nuclear factor-κB (RANK) in osteoclasts and its ligand (RANKL) released by osteoblasts [2]. In the case of a bone tumor, however, cancer cells release cytokines that tell osteoblasts to overexpress RANKL. This causes osteoclast activity to increase and bone resorption to happen because RANKL binds to RANK receptors [3].

Usually, the first approach in the treatment of bone cancer is its surgical removal, which has two main goals: (i) palliative care to relieve pain, instability, and paralysis, and (ii) tumor resection to cure the disease. The surgery for tumor removal is followed by bone reconstruction of the bone defect caused by the tumor, employing metallic and ceramic biomaterials. Chemotherapy, radiotherapy, and the use of drug protocols are employed after surgery to avoid tumor recurrence [4]. 

Bone reconstruction is an approach adopted mainly in cases of metastasis from thyroid cancer and renal cell carcinoma, as in these cases, the metastasis usually occurs in the extremities of the bone, spine, and pelvis, thereby requiring different methods of reconstruction following the tumor resection [4]. Regarding chemotherapy and drug protocols, there are two different approaches for treating bone cancer: (i) the use of chemotherapy and (ii) the use of bisphosphonates. Chemotherapeutics, such as doxorubicin and cisplatin, work as systemic drugs and are not necessarily specific for bone cancer despite their high efficacy [5]. Bisphosphonates, on the other hand, are drugs that work on osteoclasts to stop the vicious cycle that causes tumors to grow [6]. A note of caution is due here. Although pharmacological and surgical interventions are the standard approaches, they are generally intended to prolong patients’ survival or ameliorate their quality of life, as they do not necessarily lead to effective treatments; besides, patients usually suffer from disability due to the loss of functional and structural properties of their bones. This shows how important it is to find new therapies that can treat the bone and make sure it can still function [7].

In light of traditional medicine, bone reconstruction and drug protocols are approaches that have been employed individually. However, with the advance of bioceramics and drug-delivery technologies, there have been studies using bioactive ceramics as carriers of bone-cancer-target drugs, yielding a multifunctional material able to perform a bone-cancer-treatment approach with bone regeneration. The combination of cancer treatment and bone regeneration has both economic and social benefits. Because there are fewer clinical interventions, patients spend less time in the hospital, which lowers the cost of the whole treatment and improves the patient’s quality of life [8].

Examples of bioceramics used for such purposes include calcium phosphate ceramics and bioactive glasses for the delivery of drugs, chemotherapeutics, and ions with antitumor activity [9,10]. Calcium phosphate and bioactive glasses have been used as biomaterials for more than 50 years, and are suitable materials for bone regeneration, besides both being genetic names of a group of compounds. Calcium phosphates are a group of ceramics that encompasses tricalcium phosphate, hydroxyapatite, calcium pyrophosphate, dicalcium phosphate dihydrate, octacalcium phosphate, and biphasic calcium phosphate [11]. However, hydroxyapatite, tricalcium phosphate, and their combination (biphasic calcium phosphate) are the most commonly used biomaterials for bone regeneration. Bioactive glasses and glass ceramics, in contrast, cover a large class of silicate, phosphate, and borate glasses and glass ceramics that are bioactive and biocompatible. Bioactivity refers to the ability of these glasses to bond to the bone tissue like synthetic hydroxyapatite does, while biocompatibility is the ability of the glass to be placed in host tissue and trigger only a minimal immunological response, which is not pathological. To load and deliver drugs in the host tissue, calcium phosphates, bioactive glasses, and glass-ceramics have all been used. If you want to learn more about how calcium phosphates are used to treat bone cancer, we suggest reading [12,13].

However, in this work, we shall focus only on bioactive glasses, given that, differently from calcium phosphate-based ceramics, the development of mesoporous nanostructures has been more explored in bioactive glasses, and this kind of microstructure has played a significant role in the development of more sophisticated drug-delivery systems. Mesoporous bioactive glasses (MBG) nanoparticles allow for maximizing drug load, and can eventually be internalized by cancer cells, enabling target-therapy approaches. As glasses lack a long-range ordered structure, they can be doped with almost all the elements of the periodic table. This is an advantage when compared with crystalline calcium phosphate, as crystalline materials have limitations regarding doping concentration and electronic configuration. Therefore, this work reviewed the current challenges and opportunities in bone-cancer treatment by combining bioactive glasses and bone-cancer-target drugs. 

Other works from the literature have already reviewed the use of bioactive glasses in cancer treatment. However, these works were not only focused on cancer-target drugs but also included a revision on magnetic hyperthermia, brachytherapy, and photothermal therapy [14,15]. Thus, the information about drug-delivery approaches was not comprehensively reviewed. The importance of this review is due to the increasing number of articles reporting on the treatment of bone cancer but using different pharmacological approaches that are related to distinct biochemical pathways. In this way, it is relevant to put the different approaches into groups and point out their pros and cons, as well as any difficulties that still need to be solved.

## 2. Methodology

We developed a methodology to conduct this study to cover the most current developments of bioactive glasses in drug delivery for cancer treatment. A search was conducted using the terms “drug” and “drug delivery” or “controlled release” and “cancer” in the PubMed, Scopus, and Web of Science databases. We looked at articles published since 2000 to obtain the most up-to-date information on bioactive glasses used as drug carriers. Following this analysis, only papers that addressed the use of bioactive glasses in cancer treatment were considered for this review. To do so, the following keywords were used as restrictions: “bioactive glass, bioactive glass-ceramic, bioglass, and resorbable glass” in the aforementioned databases. In the first section of this review, we cover the different classes of chemotherapeutic drugs used in the management of bone cancer. Later, we will report on the barriers involved in effective chemotherapeutic approaches. Finally, we address how the use of delivery based on bioactive glasses can overcome some of the limitations involved in drug and gene delivery, chemotherapeutic administration, and bioinorganic approaches.

## 3. Chemotherapy in Cancer Treatment: Targeted Therapies

In 2021, oncological molecular entities represented 30% of the newly approved drugs by the Food and Drug Administration (FDA). In the same year, the FDA approved 50 new molecular entities, of which 36 were chemical and 14 were biologics. Most of these molecular entities are small molecules, followed by antibodies and non-antibody drugs [16,17]. The term “cancer-targeted therapy” refers to drugs specifically designed to interfere with a molecular abnormality present in tumor cells or in the tumor microenvironment that has a critical role in cancer growth and survival. Thus, this approach is recognized for improving selectivity while reducing adverse side effects. 

**Figure 1:**
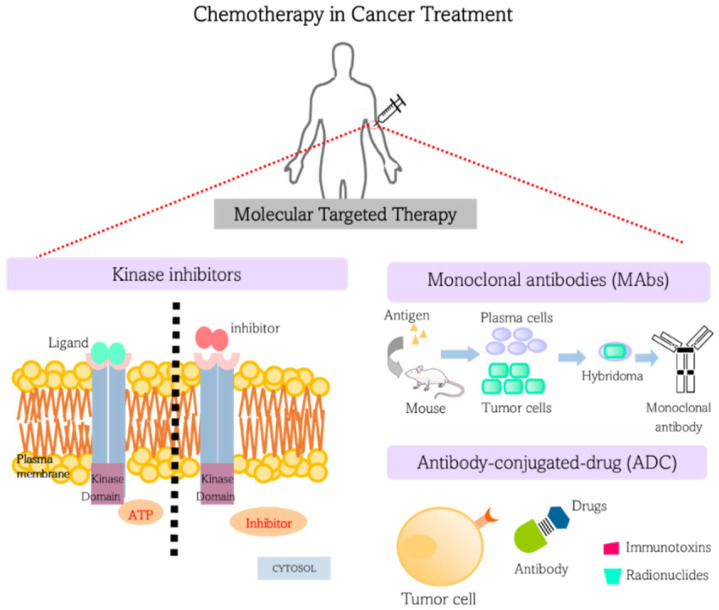
Different strategies of chemotherapy based on molecular target therapies.

Most of these drugs are categorized into two groups: (I) small molecules chemically synthesized, usually with low molecular weight and a high rate of cell entry, designed to interfere with intracellular molecules; and (II) biological molecules such as monoclonal antibodies (MAbs), which have high binding affinities to extracellular domains of cell surface receptors or soluble extracellular antigens [18]. Figure 1 schematically shows the different strategies of molecular target therapies. Most of the core molecular targets used for the design of anticancer drugs are cell surface receptors, signal transduction constituents, transcription factors, ubiquitin-proteasome proteins, and tumor microenvironment components, such as angiogenic factors [19]. In the next subsections, we shall cover the mechanism of action of some of these chemotherapeutic classes, such as: (i) kinase inhibitors; (ii) monoclonal antibodies; and (iii) antibody-conjugated drugs.

### 3.1. Kinase Inhibitors

The main targeted drugs currently approved for clinical use are protein kinase inhibitors. Usually, small molecules compete for the ATP binding sites of protein kinase in its active or inactive structure [20]. The importance of kinases as cancer targets is not without precedent since several human cancers are associated with dysfunction or overexpression of protein and lipid kinases or their regulators [20]. Currently, many protein kinases are targets of drugs used in cancer treatment, and a significant number of kinase-target drugs are in clinical or preclinical trials, indicating that kinases are key molecules for anticancer drug development [20]. Among all protein kinases, growth factor receptor tyrosine kinases (RTKs) are the most successful class, with a significant number of kinase inhibitors already being FDA-approved [21]. Besides RTKs, other kinases and non-kinase inhibitors have been developed to target other cellular regulators, including cell-cycle proteins, such as cyclin-dependent kinases (CDKs), PARP (poly ADP-ribose polymerase), proteasomes, apoptosis inducers, and DNA repair mechanisms [22].

Kinase inhibitors have been showing promising clinical outcomes, even though patient response can exhibit contrasting effects among individuals and across patient populations [23]. In addition, although treatment is usually well-tolerated, some patients might experience multi-organ toxicity with alterations in thyroid function, bone metabolism, linear growth, gonadal function, fetal development, adrenal function, and glucose metabolism, resulting in treatment discontinuation [24]. Nonetheless, because the positive effects have been more prominent than the negative ones, kinase inhibitors are still considered an effective treatment, depending on the cancer type and patient conditions.

### 3.2. Monoclonal Antibodies (MAbs)

Since the discovery of hybridoma technology by Koller and Milstein in 1975 [25], the production of mouse MAbs has transformed numerous biological areas, including cancer biology. It is now possible to produce chimeric and/or humanized immunoglobulins with minimal host immune response to antibodies through genetic engineering techniques, allowing their use in anti-tumor therapies [26]. Rituximab (directed against CD20 antigen in B cells) was the first FDA-approved MAb to enter the clinics in 1997, followed by trastuzumab (against receptor tyrosine kinase Her-2) in 1998 [27]. Nowadays, more than 30 therapeutic MAbs are approved by the FDA for cancer treatment [28]. Although most anti-cancer MAbs target tumor antigens, the most successful and promising are those designed to target and block immune checkpoints to amplify the anti-tumor T-cell response [29]. For instance, ipilimumab was the first immune checkpoint blockage MAb approved by the FDA in 2011 and was designed to target CTLA-4 (cytotoxic T lymphocyte antigen-4). Ipilimumab showed promising results in melanoma patients and remained in clinical trials for use in other tumor types [30]. In addition to ipilimumab, new MAbs targeting immune checkpoint molecules, such as programmed death receptor-1 (PD-1) (nivolumab and pembrolizumab) were also FDA-approved for the treatment of different types of malignancies [31,32,33]. We recommend checking out the nice review from Zahavi et al. [28] for a complete list of FDA-approved MAbs for cancer treatment.

### 3.3. Antibody-Drug Conjugates (ADC)

The specificity of MAbs directed against tumor antigens is also advantageous for delivering cytotoxic compounds inside the cell. This fact is the foundation of ADC (antibody-drug conjugates), where MAbs are covalently linked to cytotoxic agents, such as s drugs, immunotoxins, and radionuclides. Following antibody binding to cell-surface molecules, the cytotoxic agent is released through receptor-mediated endocytosis, resulting in cell death induction [34]. Currently, there are 10 ADCs approved by the FDA for use in cancer treatment. The first was brentuximab vedotin in 2011, a MAb conjugated with a microtubule-destabilizing drug (monomethyl auristatin E) designed to target CD30 expressed in lymphoma cells from Hodgkin and large anaplastic lymphomas [35]. In 2019, three more ADCs were approved for cancer treatment: polatuzumab vedotin-piiq (Polivy) for refractory diffuse B-cell lymphoma; enfortumab-ejfv (Padcev), and fam-trastuzumab deruxtecan-nxki (Enhertu) for metastatic breast cancer [36]. On the other hand, the delivery of biological toxins via ADC has proven difficult due to high toxicity in patients [37]. The only toxin-conjugated MAb FDA-approved is moxetumomab, a CDC-20-targeted MAb conjugated with pseudomonas exotoxin used to treat hairy-cell leukemia patients. Immunoradiotherapy with radiolabeled antibodies has also been associated with systemic toxicity and low penetration capacity in solid tumors [38]. To date, the FDA has approved two radiolabeled MAbs that target CDC20 in lymphoma cells (yttrium-90-ibritumomab tiuxetan and iodine-131-tositumomab) [39].

Currently, antibody-based medicine is the leading product in the biopharmaceutical market, with sales expected to reach USD 172.8 billion in 2022, which represents 20% of the global pharmaceutical market [40]. In 2019, 79 novel antibody-based medicines were undergoing evaluation in the late stages of clinical trials. Among these, 40 are related to cancer treatment, with the potential that 9 of them may reach regulatory review in 2022 [41]. Undoubtedly, immunotherapy has significantly impacted cancer treatment, and its efficiency has translated into better results compared to traditional chemotherapies, suggesting that MAb-based cancer therapy is a promising tool for cancer treatment [42]. Despite MAbs’ full potential, their efficacy is still relatively modest in most cases, requiring continued optimization to identify novel targets and foster investigations of how these agents could be integrated with different classes of targeted agents to provide more robust benefits.

## 4. Barriers Affecting Drug Delivery in Cancer Treatment

It is well-accepted that biodistribution is the main challenge of cancer drug delivery. On the one hand, the systemic distribution of anticancer therapies may cause many undesired side effects, with devastating consequences for the patient. On the other hand, targeting solid tumors is not easy, as they present poorly organized and leaky vasculature along with abnormal lymph vessels, contributing to high interstitial fluid pressure (IFP). Although it has been postulated that nanoparticle accumulation could benefit from the enhanced permeability and retention (EPR) effect to accumulate in some tumor tissues, this effect has not been proven reliable in clinics compared to animal models [43]. Thus, the development of a nanotechnological platform should consider its ability to diffuse against the pressure gradient. Another critical barrier to overcome is the tumor stroma, consisting of various cell types embedded in a complex extracellular matrix (ECM) [44]. Therefore, biodistribution is a major task, and achieving therapeutic concentrations of the drug in tumor tissues without damaging sensitive organs is the challenge of successful therapy. In the following subsections, we will discuss some important factors affecting biodistribution in tumor tissues.

### 4.1. Enhanced Permeability Retention (EPR) Effect

An important observation leading to the EPR effect’s characterization was that macromolecules and lipids selectively permeate the tumor vasculature, remaining in the tumor interstitium for a considerable time [45]. This fact is related to the enhanced vascular permeability of solid tumors, thus facilitating the transport of macromolecules [46]. The discovery of the EPR effect revolutionized the area of nanomedicine, and since the group of Maeda published the first anticancer nanomedicine based on the EPR concept [47,48], many researchers have proposed new EPR-based anticancer nanosized platforms [46,49,50]. The EPR effect is based on the molecular size, with particles larger than 40 kDa showing a prolonged circulatory half-life and a higher area under the concentration–time curve (AUC) [51]. Macromolecules larger than 40 kDa are above the renal filtration threshold, limiting clearance, and accumulating in tumor tissue for an extended period [52].

Interestingly, the size, shape, and surface characteristics are essential to alter particle biodistribution and tumor retention [53]. Furthermore, several other factors have been proposed to influence the EPR effect, which can be useful in clinics [46]. Although the EPR effect created new expectations for the development of selective anticancer agents, translation to clinics does not go at the same pace. Several factors contributed to the slow clinical application of nanomedicines, including the particle’s physicochemical characteristics, which should consider the factors that would influence its intracellular internalization, its unspecific interaction with the ECM, its recognition by the reticuloendothelial system (RES), and its biocompatibility and release rate. Tumor tissues present significant heterogeneity, i.e., diverse tumor types of various sizes and with different vascular abnormalities [50,54], which contributes to affecting EPR effect-based nanomedicine efficiency.

### 4.2. Protein Corona Effect

Another critical effect affecting biodistribution is protein corona, which coats particles with serum proteins [55]. Because of the nanoparticle coating, slight alterations in surface charges are expected, leading to further interactions between serum proteins in a cascade fashion and thus preventing the interaction between nanomedicines and their tumor targets [56]. However, it is essential to emphasize that the protein corona effect involves two classes of components affecting nanomedicines’ biodistribution: opsonins and dysopsonins [57]. Opsonins, such as proteins of the complement system (C3b and C1q) and immunoglobulins, are the most common, and their adsorption on the surface of nanoparticles is responsible for their engulfment and clearance by the RES-associated phagocytic cells, more specifically in the liver and spleen [58,59,60,61,62,63]. Engrafting hydrophilic or amphiphilic polymers, such as poly(ethylene glycol) (PEG), on the surface of the particles, is one strategy for reducing RES recognition [60,64,65]. Despite the popularity of this technique, issues related to PEG molecules’ stability and immunogenicity have emerged in the last few years [66,67]. Recently, zwitterionic materials, such as sulfobetaine, have been used to confer stealth properties. Their mixed charges and electrostatic repulsion account for dense packing, thus impairing protein corona formation [68,69,70,71]. In contrast, dysopsonins, such as clusterin (apolipoprotein J) and albumin, have the opposite effect, prolonging nanomedicine’s half-life in circulation [64,72]. For example, the glycoprotein CD47, which is expressed in all cellular membranes of humans, mice, and other mammals [73], interacts with CD172a on phagocytes [74] and inhibits macrophage uptake of antibody-coated mouse red blood cells (RBCs) [75]. Based on these facts, Rodriguez and colleagues demonstrated that pre-adsorption of CD47 minimizes the phagocytosis of nanoparticles, thus increasing delivery [76].

### 4.3. High Interstitial Fluid Pressure (IFP)

The interstitial space presents several vital activities, such as nutrient and oxygen transport [77]. Whereas a negative transcapillary pressure gradient arrives in normal tissues, contributing to an outward flow, in cancer tissues, a higher IFP can be evidenced, constituting a barrier for therapeutics to overcome [78,79]. Small molecules usually experience a diffusion flow, traveling from the highest to the lowest concentration gradient. In contrast, nanoparticles’ movement is associated with convection, which is pressure-dependent [79,80]. In this case, the uptake of nanoparticles is inhibited by the high IFP, and less than 1% of administered nanoparticles reach cancer cells [81]. In addition, it has been demonstrated that tumors have altered clearance times due to an absence of lymphatic drainage, low interstitial hydraulic conductivity, and great interstitial transport distances [82]. Moreover, a disorganized vascular basement membrane and associated pericytes also contribute to the variability in blood flow and drug distribution, leading to heterogeneous drug delivery in tumor tissues [83,84].

The imbalance in cellular and ECM composition of the tumor is another factor that contributes to the high IFP. Tumors are intrinsically stiffer than the healthy tissue surrounding them [85,86]. Stiffening is associated with a favorable increase in ECM deposition against its degradation, even considering the upregulation of matrix metalloproteinases (MMPs), which degrade ECM. Thus, it is expected to increase the number of matrix proteins in the tumor environment. Hypoxia and transforming growth factor- (TGF-) are two factors that stimulate ECM production, which contributes to IFP [87,88]. Increased tumor cell density also induces tumor stiffening in several ways [88]. One crucial component is related to solid pressure, which arises from the increased tumor mass. Both the formation of insoluble biomass in the tumor and stromal cells and the absorption of water by glycosaminoglycans, such as hyaluronan, expand the tumor mass, leading to compressive stress by the resistance imposed by the surrounding tissue [89,90]. Solid stress also affects the blood and lymphatic vessel structures, resulting in disturbed fluid entry and exit [90]. Therefore, the increase in solid stress limits drug distribution by causing low perfusion, which may also induce drug resistance by rendering the tumor microenvironment hypoxic and acidic [91,92,93]. In addition, fluid retention resulting from lymphatic compression results in reduced interstitial transport [93,94]. In conclusion, this solid pressure imposed by tumor hyperplasia and the overproduction of extracellular proteins impairs vascular and interstitial transport.

### 4.4. Tumor Stroma

The tumor stroma is formed by the neoplastic cells and various stromal and inflammatory cells interconnected with their ECM, which helps stiffen the tissue, thus decreasing therapy diffusion and, consequently, efficacy [88]. For instance, it has been shown that the poor prognosis of pancreatic ductal adenocarcinoma (5-year survival < 5%) is related to the dense stroma, which inhibits drug penetration [95]. Stromal cells can support tumor growth in different ways, including the induction of immunosuppression, angiogenesis, and ECM remodeling. These effects require the interaction between cancer cells and the microenvironment, which may occur directly through cell–cell contact or indirectly by the secretion of soluble factors and extracellular vesicles [96]. In addition to its role in cancer progression and metastasis, the tumor stroma also significantly impacts drug sensitivity by inducing alterations in the molecular networks operating in cancer cells [97]. These include the modulation of anti-apoptotic and oncogenic pathways associated with survival [97,98,99]. Among the stromal cells, cancer-associated fibroblasts (CAFs) are an abundant population of cells that facilitate tumor proliferation by producing ECM and impairing drug delivery. These cells can express membrane receptors, functioning as a barrier along the blood vessels for drug absorption [100,101].

To overcome stromal barriers, stromal-rupturing agents have been used before or in combination with chemotherapy. Examples of these agents are quercetin, a Wnt16 inhibitor, which can reduce fibroblasts and collagen production [102], and LY364947, a TGF-β type I receptor inhibitor, which decreases fibrosis [103]. Another strategy has been tested to address the potential of proteases to digest ECM components, thus facilitating stromal penetration. This is the case of collagenase and hyaluronidase, which can be delivered in nanoplatforms [104,105]. However, one might consider that components of the ECM may stimulate tumor growth once they are cleaved into small fragments, functioning similarly to growth factors [106,107]. Therefore, nanoplatforms’ design to achieve penetration into the tumor stroma should be carefully considered to improve efficacy [108].

## 5. Bioactive Glasses in Drug Delivery Applied in Cancer Treatment

Bioactive glasses have been used in bone regeneration since the 1970s after being developed by L.L. Hench. Originally, these glasses were based on the system 45SiO_2_-24.5Na_2_O-24.5CaO-6P_2_O_5_ (wt.%), which is well-known as 45S5 Bioglass^®^, but currently there are glasses based on the ternary (SiO_2_-CaO-P_2_O_5_) and binary (SiO_2_-CaO) silicate system, as well as other systems based on borate and phosphate glasses [109,110,111]. Because of a mechanism known as bioactivity, these glasses share the ability to chemically bond to hard and soft tissues when used as implants or scaffolds. It is a series of surface reactions that occur at the interface between the glass and the body fluid that ultimately leads to the formation of hydroxyapatite on the glass surface. Briefly, the mechanism of bioactivity of silicate glasses is described in five steps, although the formation of hydroxyapatite in borate and phosphate glasses happens similarly: (1) diffusion of H^+^ species from the body fluid towards the glass surface, resulting in glass modifier leaching; (2) cleavage of Si-O-Si bonds that results in the formation of silanol (Si-OH) bonds; (3) intensification of Si-O-Si bond cleavage, and formation of a silica-gel layer on the glass surface; (4) Ca^2+^ and PO_4_^3−^ ions precipitate onto the silica-gel layer, forming an amorphous calcium phosphate layer; (5) the amorphous calcium phosphate layer crystallizes into hydroxyapatite [110,111,112].

Because of the bioactivity mechanism, bioactive glasses have been used in biological applications that require regeneration, such as bone regeneration, wound healing, dental repair and regeneration, and peripheral nervous system regeneration, among others. Therefore, it is natural that most of the bioactive glasses applied in bone-cancer treatment focus on combining bone regeneration with cancer treatment. In this case, the glasses can be loaded with compounds or ions with anti-cancer properties that are delivered to the cancer site, working as a drug-delivery system. However, bioactive glasses are not restricted to being applied in bone cancer treatment by carrying bone-cancer target drugs; technologies involving magnetic bioactive glasses and radioactive glasses have also been developed for applications in magnetic hyperthermia and brachytherapy, respectively (please see refs. [113,114,115] for detailed information about bioactive glasses applied in magnetic hyperthermia and brachytherapy). Multifunctional bioactive glasses that combine magnetic hyperthermia or brachytherapy with drug delivery have also been proposed, as will be covered throughout this review.

**Figure 2:**
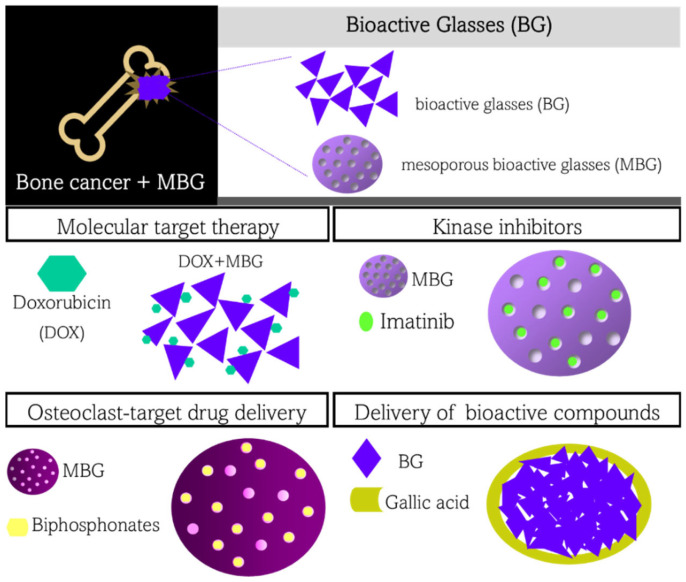
Application of bioactive glasses as drug carriers of drugs used in bone cancer treatment.

Even though most chemotherapeutic drugs are classified into molecular target therapy, kinase inhibitors, monoclonal antibodies, and antibody-drug conjugates, bioactive glasses have been mostly used as drug-delivery carriers of either molecular target therapy or kinase inhibitors. Besides these classes of drugs, bioactive glasses have been used as carriers of other drugs applied in bone cancer treatment, such as bisphosphonates, which are drugs that act on the osteoblasts’ metabolism. Other examples, such as mRNA, lysosomes, and inorganic ions (therapeutic ions) delivery, do not fall into any other category and were considered by us as bioactive compounds and therapeutic ions. In the next sections, we summarize the applications of bioactive glasses in each drug category, including (i) molecular target therapy, (ii) kinase inhibitor, (iii) bisphosphonates, and (iv) bioactive compounds and therapeutic ions. It is worth noting that most of the bioactive glasses used as drug-delivery systems for cancer treatment are based on mesoporous structures (the well-known mesoporous bioactive glasses, MBG). The advantage of using mesoporous glasses is that drugs can be loaded within the mesoporous, diminishing their accessibility, thereby promoting the improved controlled release desirable in drug delivery systems to keep the drug within the therapeutic window. Figure 2 schematically shows how bioactive glasses, or MBG, can be used as a carrier of drugs applied in cancer treatment.

### 5.1. Bioactive Glasses for Delivery of Molecular Target Therapy

Among the classes of chemotherapeutics used in bone cancer treatment, the delivery of molecular target therapy is the most explored strategy in combination with bioactive glasses. Because doxorubicin (DOX) is a well-known and effective chemotherapeutic for cancer treatment despite its low specificity, it has been one of the most researched drugs in combination with bioactive glasses. However, other well-known chemotherapeutics have also been explored, such as mitomycin C and methotrexate. We have summarized the most recent advances in the aforementioned chemotherapeutics below.

**Doxorubicin (DOX):** among the compounds used in molecular target therapy, doxorubicin is the most commonly used drug. Doxorubicin binds to DNA double strands, impeding DNA transcription and thereby affecting protein production and cell function. Additionally, the drug can induce the generation of free radicals and oxidative damage to biomolecules. Drug-delivery systems based on bioactive glasses have been combining the delivery of DOX with (i) magnetic hyperthermia, (ii) compounds that improve bone regeneration, and (iii) stimuli-responsive systems.

(i) DOX + magnetic hyperthermia: Vernè et al. [116] were the first to report the use of magnetic hyperthermia in conjunction with anticancer drugs such as DOX and cisplatin. It was reported that cisplatin and DOX could bind to OH groups on the glass-ceramic surface, favoring very slow-release kinetics. Zhang et al. [117] proposed 3D-printed scaffolds based on Fe_3_O_4_/MBG/PCL and showed a controlled release of DOX from the mesoporous scaffold. Additionally, the polymeric phase of PCL favors a more sustainable release. Both materials were designed for dual therapy, combining the benefits of chemotherapy with the enhanced drug permeability and hyperthermic effect provided by magnetic hyperthermia. However, none of these studies [116,117] performed in vitro tests to evaluate the effect of DOX and cisplatin on bone cancer and healthy cells. Therefore, it is not possible to evaluate whether magnetic hyperthermia and the amount of DOX released by these drug-delivery systems were able to show a synergetic effect to treat cancer, nor to evaluate their effect on bone regeneration. 

(ii) DOX + compounds or ions that promote bone regeneration: As previously stated, DOX and other drugs can bind to the surface of the MBG, allowing for controlled release. However, different drugs display different strengths of physical–chemical interactions (adsorption or intermolecular interaction), which alter the drug release kinetics. Czarnobaj et al. [118] took advantage of that fact and proposed MBG as carriers of DOX and metronidazole; the latter is an anti-inflammatory drug that could favor bone regeneration by avoiding severe inflammation in the cancer-treated area. Due to the presence of more hydroxyl and amine groups in its molecular structure, the DOX has slower release kinetics than metronidazole in this case. By releasing metronidazole faster than DOX, it might have an anti-inflammatory effect on the biomaterial during the cancer treatment, but more in vitro and in vivo studies are needed to see if this is true.

Besides the delivery of organic molecules, the delivery of therapeutic ions can also be performed to favor bone regeneration, which is a treatment based on bioinorganic concepts. For example, rare earth elements, such as Sm^3+^, Yb^3+^, Tb^3+^, and Eu^3+^ can be used to stimulate the bone regeneration response once they have a high affinity for calcium sites in biological molecules, either acting as Ca^2+^ inhibitors or probes. Additionally, these rare earth elements can modulate DOX release kinetics in drug-release studies, although they affect DOX release differently [116,119,120]. When MBG is doped with Yb^3+^ or Tb^3+^, the rare earth ions act as glass modifiers in the glass structure, increasing glass solubility; however, because the DOX chemically interacts with Ca^2+^ and Yb^3+^ in a chelation mechanism, the DOX release is controlled by the chelation affinity rather than the glass dissolution. In this sense, Yb^3+^ and Tb^3+^ display higher chelation affinity, decreasing the release kinetics of DOX. On the other hand, the incorporation of Sm^3+^ in the glass structure yields higher release kinetics of DOX from the mesoporous, suggesting a lower chelation affinity of Sm^3+^ compared to Ca^2+^. In contrast, Zhang et al. [119] reported that during the glass synthesis, Eu^3+^ was responsible for modulating the pore size of the mesoporous structure in the glass, consequently affecting DOX release. Therefore, instead of a chelation mechanism, Eu^3+^ had a direct influence on the microstructure of the drug carrier, promoting a physical barrier for drug accessibility rather than a chemical barrier. Zhang et al. also reported a possible synergetic cytotoxic effect of DOX and Eu^3+^ on osteosarcoma MG-63 cells, which would be an advantage of their drug-delivery system. In Section 5.4, we further discuss the effect of rare earth on cancer and healthy cells. Altogether, these results are interesting because they highlight the possibility of using a rare earth element to modulate DOX release.

(iii) DOX + stimuli-responsive systems: By functionalizing MBG, the surface structure can be changed to make them stimuli-responsive. Polo et al. [121] loaded the mesopores of MBG with DOX, functionalized the glass surface with triamine bonds, and capped them with ATP (adenosine triphosphate) molecules. In this sense, the ATP molecules on the surface function as alkaline phosphatase (ALP) stimuli-sensitive gates. When there is a high concentration of ALP in the microenvironment, such as in osteosarcoma, the ATP is broken down by enzymes, letting the DOX out of the mesopores. The authors evaluated their system in human osteosarcoma cells (HOS) and showed that cell viability was lower when the culture medium was supplemented with ALP to simulate a tumor microenvironment. However, the MBG only displays bioactivity after opening the molecular gate by ATP enzymatic cleavage. Aina et al. [122] showed that the surface of bioactive glasses could be functionalized with APTES (aminopropyltriethoxysilano) bonded to a maleamic acid; the latter can be further bonded to a DOX-like molecule. The chemical bond between maleamic acid and DOX is affected by acidic pH (pH 5.0) and the pH of tumor environments. Thus, DOX is selectively delivered into the cancer microenvironment.

**Methotrexate:** It is a molecule that binds to and inhibits dihydrofolate reductase, affecting the folic acid production needed for DNA synthesis. Chen et al. [123] functionalized the surface of glasses with folic acid and methotrexate. Folic acid functionalization was done because tumor cells overexpress folic acid receptors responsible for endocytosis, making them a molecular target for anti-cancer therapies. Thereby, by functionalizing the glass surfaces with folic acid, the glasses could be internalized by the cancer cell and deliver methotrexate within the cytoplasm, enhancing its anti-tumor property. In fact, in vitro results showed that those samples containing methotrexate showed cytotoxicity toward HeLa cells, while samples containing only folic acid or no functionalization did not show the same effect. These findings emphasize the significant role of surface functionalization on drug delivery mechanisms, which can be further explored with other drugs. Strategies similar to the use of folic acid can also be used to overcome the barriers involved in cancer treatment, such as tumor stroma, high interstitial fluid pressure, and the protein corona effect.

**Mitomycin C**: It is a drug that crosslinks DNA strands and inhibits their transcription. Rahman et al. [124] loaded magnetite-containing MBG nanoparticles with mitomycin C. They demonstrated that such drugs are specifically cytotoxic to MG-63 cancer cells (osteosarcoma cells), whereas normal human fibroblast cells (NHFB cell line) do not show significant cytotoxicity. Additionally, because of the superparamagnetic properties of the composite nanoparticles, they could be used for hyperthermia applications, thereby being considered dual-therapy platforms for cancer treatment. In another study, Shoaib et al. [125] also proposed a dual-therapy drug delivery system based on mitomycin C and MBG nanoparticles. However, they combined chemotherapy for cancer treatment with bone regeneration triggered by Mn ions doped in the glass structure. In this sense, Mn is considered a therapeutic ion. Similar to Rahman et al. [124], Shoaib and colleagues reported that their drug-delivery system exhibited selective cytotoxicity toward MG-63 osteosarcoma cells while not affecting NHFB cell viability.

### 5.2. Bioactive Glasses for Delivery of Kinase Inhibitor

Bioactive glasses have been poorly explored as drug-delivery carriers of kinase inhibitors. So far, only one study has shown their potential to carry a kinase inhibitor. Shoaib et al. [125] loaded mesoporous nano-bioactive glasses with imatinib and showed their potential against MG-63 (osteosarcoma) cells. Imatinib is a tyrosine kinase inhibitor since it binds to an ATP-binding site of the enzyme, blocking its activity, avoiding tyrosine phosphorylation, and yielding the blocking of tumor cell proliferation. MBG-containing imatinib showed high cytotoxicity against MG-63 cells, but the studies were not carried out with healthy cells, restricting further comments about its specificities. Interestingly, imatinib-loaded MBG showed different drug-release kinetics at different pH. About 50% of imatinib was delivered at alkaline or neutral pH after ten days in in vitro experiments. In contrast, at acidic pH, about 80% of imatinib was delivered in the same period. This fact emphasizes that the drug-delivery system has better outcomes regarding imatinib delivery into the tumor microenvironment. 

According to Palmerini et al. [126], kinase inhibitors have shown effective results in rare bone cancers, such as vascular tumors, malignant solitary fibrous tumors of bone, and synovial sarcoma. Thereby, new studies focusing on kinase inhibitors are encouraged in the future.

### 5.3. Bioactive Glasses for Delivery of Osteoclast-Target Drugs

A disequilibrium between osteoblast matrix deposition and osteoclast bone reabsorption favors bone tumor growth. To appreciate such a relationship, an understanding of osteoclastogenesis is needed. Osteoclasts are derived from hematopoietic stem cells, and their differentiation into osteoclasts is dependent on molecule signaling, including the receptor–activator of NF-B ligand (RANKL), which is expressed by osteoblasts. Differentiated osteoclast cells resorb the bone matrix through acidic bone demineralization. The release of growth factors trapped in the bone matrix, such as TGF-β, IGFs, and Ca^2+^, which act as signaling molecules in osteoblasts, occurs during this process, and the relationship between RANKL and these growth factors is one of the facts related to healthy bone homeostasis. However, these growth factors also stimulate bone tumor growth, which, in turn, releases other growth factors that stimulate the differentiation of hematopoietic cells into osteoclasts. Therefore, it generates the so-called “vicious cycle,” once osteoclast activity induces bone tumor growth and bone tumors induce osteoclastogenesis [3].

Clinical therapeutic strategies have focused on using drugs to stop the vicious cycle, such as bisphosphonates, a class of drugs grouped into non-nitrogen-containing and nitrogen-containing molecules with a chemical structure similar to pyrophosphate. Bisphosphonates have high specificity for bone tissue, as they bind to the hydroxyapatite of the bone and are absorbed by osteoclasts during bone resorption. After cellular uptake, non-nitrogen-containing bisphosphonates are incorporated into newly formed adenosine triphosphate (ATP) by class II aminoacyl-transfer RNA synthase, generating non-hydrolyzable ATP that inhibits ATP-dependent cellular processes. On the other hand, nitrogen-containing bisphosphonates bind to and inhibit the farnesyl pyrophosphate synthase, disturbing the mevalonic acid pathway that is responsible for the synthesis of several important biomolecules, such as cholesterol, other sterols, and lipids [127]. Ultimately, both non-nitrogen-containing and nitrogen-containing bisphosphonates lead to osteoclast apoptosis. Consequently, the vicious cycle is stopped, leading to tumor regression. Recent studies have even shown that bisphosphonates can also inhibit bone metastasis. Popular bisphosphonate drugs include risedronate, alendronate, and zoledronic acid [128].

Bioceramics have been used with bisphosphonates because they are also used in osteoporosis treatment once they block osteoclast activity, which is also desired in osteoporosis conditions. However, only a few studies have mentioned the use of bioactive glasses in conjunction with bisphosphonates to treat bone cancer. Boaninit et al. [129] loaded mesoporous bioactive glass nanospheres with alendronate at different concentrations up to a maximum of 17 wt% and showed that this drug-delivery system could be a potential tool for bone tumor treatment. In in vitro models, this drug-delivery system reduced osteosarcoma cell viability (MG-63) to 25% after 7 days. Alendronate-loaded nanospheres were able to avoid the differentiation of a murine monocyte/macrophage cell line (RAW 264.7) into an osteoclast in a conditioned medium supplemented with RANKL, suggesting that the alendronate was able to be released and inhibit RANKL receptors. In another study, Valimaki et al. [130] showed that a bioactive glass microsphere loaded with zoledronic acid led to the favorable remodeling of the tubular bone structure in bone fracture regeneration in in vivo models using Sprague-Dawley rats. These findings provide biological evidence that bisphosphonate use may have an anti-tumor effect as well as promoting bone regeneration, acting as a two-in-one drug.

In contrast with these aforementioned works, a study has produced bioactive glasses containing alendronate [131], intending to increase bone matrix mineralization by promoting osteoinduction and osteoconduction while limiting osteoclast activity. However, this work did not find a synergistic effect between the glass and the bisphosphonates, but rather a reduction in the drug’s effect on bone resorption. The strong chemical affinity between the phosphate moiety of bisphosphonates and calcium sites on bioactive glass surfaces may explain the loss of bisphosphonate activity when used with bioactive glasses. Chemically bonded bisphosphates formed through electrostatic interactions have different release kinetics than those chemically adsorbed on the glass surface, thereby partially inactivating the bisphosphonate [132,133].

Recently, our research group overcame the limitation of combining bisphosphonates with bioactive glasses by developing a thermoreversible hydrogel based on Pluronic F127, containing Ho-containing bioactive glasses, and zoledronic acid, aiming for bone cancer treatment by brachytherapy (^166^Ho) and bisphosphonate action. We showed that by modulating the concentration of zoledronic acid in the hydrogel, it was possible to encapsulate part of the compound (which was named free zoledronic acid), while another portion of the drug was bonded to the glass surface (named bonded zoledronic acid). At a certain concentration of zoledronic acid in the hydrogel (2.5 mg mL^−1^), the concentration of free zoledronic acid was enough to perform selective cytotoxicity on osteosarcoma cells (MG-63), while still favoring in vitro bone regeneration and biocompatibility toward pre-osteoblast cells (MC3T3-E1) [134].

Even though there is not much written about osteoclast-target drugs and bioactive glasses, the results so far are very promising, and more research should be conducted because of how well they work against the vicious cycle.

### 5.4. Bioactive Glasses for Delivery of Other Bioactive Compounds and Therapeutic Ions

This review used the term “bioactive compound” to refer to any biomolecule or inorganic ion that shows biological activity against cancer cells and does not fall into any previous categories. Below, we present these strategies for bone cancer treatment.

**Gallic acid:** Gallic acid is a polyphenol found in some higher plant families that exerts antitumoral properties through different pathways. Gallic acid can either activate caspases that regulate apoptosis or generate ROS (reactive oxygen species) that cause cell death. The advantage of using gallic acid is its anti-tumor effectiveness, even in cisplatin-resistant cells. Ferrimagnetic bioactive glasses used in magnetic hyperthermia were coated with gallic acid and displayed an enhanced pro-oxidative effect, evidencing their potential application in cancer treatment [135].

**Gene therapy:** It is based on the intracellular delivery of genes, typically miRNA, to regulate gene expression at the posttranscriptional level by binding to mRNA UTR sequences and inhibiting protein translation. Usually, gene delivery is made by viral-based vectors, cationic polymers, or liposomes, but their lack of effectiveness falls into three categories: the high cost (for viral-based vectors), low transfection efficiency, and cytotoxicity at high concentrations (for non-viral-based vectors). In this sense, Yu et al. [136] showed that bioactive glass nanoparticles could be successful carriers of genes. Bioactive glass nanoparticles were shown to be more efficiently absorbed by bone mesenchymal stromal cells (BMSC) than polyethyleneimine (PEI 25 K), a commercial gene vector. Li et al. [137] showed that MBG nanoparticles could be dual-loaded with miRNA and DOX, and both bioactive compounds showed cytotoxic effects after nanoparticle uptake by HeLa cells. These results show that bioactive glass nanoparticles can be a potential vector for gene therapy. However, because gene therapy is still limited to the treatment of rare serious or life-threatening diseases with few treatment options, research on this therapy may focus on rare bone dysplasia diseases [138] if it is intended to make this therapy approved by health and sanitary regulation offices. 

**Inorganic ions, bioinorganic or therapeutic ions:** All of these terms refer to the same thing: the role of inorganic ions in biological systems. In any case, in the field of bioactive glasses, the term “therapeutic ions” is the most commonly used. Therapeutic ions can display different biological functions, such as increasing osteogenesis, angiogenesis, wound healing, a bactericidal effect, and even anti-tumor activity, the last of which is the focus of this review [139]. Because of the amorphous nature of glasses, bioactive glasses can be doped with almost all the elements of the periodic table, which opens a wide range of opportunities to use inorganic ions for biomedical applications using bioinorganic concepts [14].

Some ions have shown a therapeutic effect in cancer cells, including alkali-earth, transition, rare-earth, and chalcogen elements, which have been intentionally added to the glass structure to display a biological effect on cancer cells. However, before commenting on the effect of therapeutic ions on cancer cells, a note of caution is in order. Recent research has shown that bioactive glasses, including the 45S5 composition, have selective cytotoxicity against giant cell tumors of the bone-derived from neoplastic stromal cells but not stromal cells derived from bone marrow. Westhauser et al. [140] showed that the 45S5, ICIE16, and glasses doped with 3 wt.% of Mg, Zn, or B all show selective cytotoxicity, but the 45S5 showed the highest cytotoxic effect and was further analyzed in their studies. Briefly, the authors addressed a necrotic effect displayed by the 45S5 glass as the main mechanism underlying its selective cytotoxicity toward giant cell tumors. As necrotic death is related to a caspase-independent cell death mechanism, it is believed that the glasses were able to disrupt the cell membrane and cause its death.

Regarding alkaline-earth elements, Sui et al. [141] compared the anti-tumor activity of mesoporous nanoparticles of bioactive glasses (SiO_2_-CaO) and silica (SiO_2_). It was shown that those nanoparticles containing calcium activate transient receptor potential channels and calcium-sensing receptors on hepatocellular carcinoma cells (HepG2), mediate calcium influx, and directly regulate the calpain-1Bcl-2caspase-3 signaling pathway to suppress tumor growth without affecting healthy cells. Calcium sites on bioactive glass surfaces interact with DOX molecules used for drug delivery, slowing down their release kinetics and yielding less systemic toxicity than silica nanoparticles. All of these facts suggest that bioactive glass structure should be prioritized when it comes to silica mesoporous nanoparticles. Interestingly, when the work of Sui et al. [141] is contrasted with the work of Westhauser et al. [140], the selective cytotoxicity effect of the glass is displayed by very different mechanisms, that is, caspase-mediated and caspase-independent pathways, respectively. It is worth noting that Westhauser et al. [140] investigated whether caspase-mediated pathways were affected in their studies but found no interference. Considering that the authors evaluated the effect of these glasses on different cells, it is reasonable to assume that bioactive glasses may display different mechanisms of cell death depending on the tumor cell origin. However, more studies are needed to understand these mechanisms in depth.

Concerning transition metals, some of them are likely to selectively display toxic effects on cancer cells, leading to cancer cell death. Chen et al. [142] showed that zinc-containing silica mesoporous nanoparticles (containing 14 mol% of Zn) could efficiently yield breast cancer cells’ apoptosis, but not that of healthy epithelial cells. In another study, Kilcup et al. [143] showed that glasses containing V_2_O_5_ (between 2.25 and 9 mol%) and ZnO (between 11 and 20 mol%) also showed selective apoptosis of liver cancer cells, but the authors addressed the apoptotic effect of vanadium rather than zinc ions. Although it is not possible to establish a relationship between the studies of Chen et al. [142] and Kilcup et al. [143] due to the difference in the glass composition, the results of Kilcup et al. bring interesting knowledge about vanadium as a therapeutic ion, as it showed a higher cytotoxic effect on cancer cells than zinc.

Concerning rare earth elements, recently, our research group showed that Ho^3+^ ions from Ho-containing bioactive glasses display selective cytotoxicity on human osteosarcoma MG63 cell lineages while favoring high biocompatibility in pre-osteoblast-like cells (MC3T3-E1), emphasizing the selective biological effect of rare earth elements on cancer cells that can be explored in future works [7,144]. As aforementioned in Section 5.2, rare earth elements can mimic Ca^2+^ ions in biological pathways and enhance or block some biological activities. Other studies from the literature have either individually observed the cytotoxicity of other rare earth elements on osteoclasts, or individually reported the enhanced biocompatibility promoted by this class of elements. The higher biocompatibility of rare earth elements has also been noticed in glasses doped with Gd and Yb [145], Tm [146], Eu [147,148], Sm [149], Y [146], La [150], Eu and Tb [147]. Considering that rare earth-doped glasses may display potential properties for applications in bone cancer treatment by brachytherapy, they can either be used as therapeutic ions in bioinorganic applications or be neutron-activated for radiotherapy purposes, as in both cases, enhanced bone regeneration is expected due to their agonistic behavior at Ca^2+^ sites in biomolecules.

Finally, concerning chalcogen elements, Te and Se have also been used as doping elements in MBG [151,152,153,154]. Se^4+^ and Te^4+^ ions display bactericidal and anti-cancer properties as these ions have a high redox potential. When dissolved from the glass structure, these ions can be uptaken by MG-63 osteosarcoma cells, be stored and accumulated in the mitochondria, and later released in the cytoplasm, where they produce reactive oxygen species (ROS) that, in turn, cause oxidative stress and lead to cell death. Moreover, this cytotoxic effect caused by oxidative stress from Se^4+^ and Te^4+^ is more pronounced in MG-63 cells than in pre-osteoblast MC3T3 cells, evidencing a selective potential for cancer treatment. Interestingly, unlike all the aforementioned therapeutic ions, Se^4+^ and Te^4+^ are more likely to be glass formers in the glass structure than glass modifiers. However, despite the higher glass polymerization displayed by these ions, it does not affect the bioactivity of chalcogenide-doped MBG. If the chalcogenides-doped MBG are loaded with DOX, the electrostatic interaction between Se^4+^ and Te^4+^ and the drug slows down the release kinetic constant of the drug, similar to the effect of rare earth elements discussed in Section 5.2.

All the applications discussed here are summarized in Table 1. 

## 6. Conclusions

Bioactive glasses have been successfully applied in drug delivery systems as carriers of cancer-target drugs and new therapies, such as gene delivery and bioinorganic. Despite the different classes of drugs, bioactive glasses have been mainly employed in the delivery of some chemotherapeutic classes, such as molecular-target drugs and kinase inhibitors, while others remain unexplored, such as monoclonal antibodies and antibody-drug conjugates. Bisphosphonates are interesting drugs to be explored in drug-delivery systems derived from bioactive glasses and focusing on cancer treatment. Bisphosphonates favor tumor reduction and improve the regeneration of bone defects caused by tumors, but their conjugation with bioactive glasses has been mainly focused on osteoporosis. Regardless of the drug used in cancer treatment, the intermolecular interactions between the drug and the glass surface seem to be a determinant factor for improved controlled release systems. However, complementary strategies can be used, such as functionalizing the glass surface, coating the glass surface with a polymer, or producing composites based on bioactive glasses and polymers or hydrogels. Regarding the functionalization of the glass surface, the use of molecules able to bind to overexpressed receptors in osteosarcoma cells (such as folic acid) can be an interesting strategy to increase the specificity of the treatment. Concerning the production of composites made of glass, polymers, or hydrogels, the development of pH-sensitive drug-delivery systems can be another alternative to allowing drug release only at low pH, below 5, which is the typical pH of the tumor microenvironment. The glass structure can also be doped with alkali metals, transition metals, rare earth elements, and chalcogens to either favor bone regeneration or cancer treatment.

Finally, some of these strategies have been used in the literature, but there is still plenty of room for discoveries aimed at cancer treatment. For example, some of the aforementioned works have well-evaluated the physical–chemical aspects of their drug-delivery systems but did not perform in vitro and in vivo tests, which impede a critical analysis of their drug-delivery systems, despite the promising results evidenced by the physical–chemical characterization. Thereby, further studies evaluating biological aspects like treatment specificity, in vivo studies of organ toxicity, and the in vivo pairing of bone regeneration and cancer treatment are some examples of areas to be covered to determine whether these drug delivery systems can increase cancer treatment efficacy at the same time that bone regeneration may occur. Another critical point is that the development of drug-delivery systems based on bioactive glasses has been performed under an old-fashioned perspective of drug delivery, which focuses on maximizing drug loading and controlling sustained delivery. However, effective strategies to overcome the limitations of cancer treatment, such as the EPR effect, protein corona, tumor stroma, and high interstitial pressure, should be taken into account, as these strategies may lead to improved treatment and are less likely to yield drug resistance, which is a typical problem faced during cancer treatment. Moreover, these strategies are inclined to work at the nanoscale, thereby making the production of glasses by the sol-gel method, which focuses on the production of nanoparticles, of biological relevance. Considering that the advance of MBG nanoparticles is well-established in the literature and already improves drug loading and controlled release, their functionalization with molecules, such as quercetin and LY364947, aiming at overcoming the barriers caused by tumor stroma, or the functionalization with glycoprotein CD47 to override some protein corona effects, are some examples of glass functionalization yet to be explored and might lead to improved outcomes in in vivo studies.

## Figures and Tables

**Table 1:** List of main finds from the literature, cataloged in accordance with cancer therapy combined with bioactive glasses.

	Material	Glass System	Cancer Therapy	Main Finds	Ref.
Molecular Target Therapy	Melt-derived magnetic bioactive glass	24.7SiO_2_-13.5Na_2_O-13.5CaO-3.3P_2_O_5_-14FeO-31Fe_2_O_3_ (wt.%)	DOX + cisplatin + magnetic hyperthermia	Pre-treatment of the glass surface with an aqueous solution led to an increase in the hydroxyl group that, in turn, interacted with DOX and cisplatin increasing drug loading, but causing random drug release kinetics.	[116]
	Fe_3_O_3_/MBG ^1^/PCL ^2^	80SiO_2_-15CaO-5PO5 (mol%)	DOX + magnetic hyperthermia	DOX was loaded into the mesopores of the MBG glass agitation of the glass particles in solution, yielding an 84.8% loading efficiency. Later, the DOX-loaded MBG was 3D-printed together with Fe3O4 and PCL. The resulting scaffold showed controlled release properties.	[117]
	MCM-41	SiO_2_	DOX + metronidazole	DOX and an anti-inflammatory drug were loaded into the mesopores. The competitive release between DOX and metronidazole was modulated by the number of polar moieties in the drug molecular structure. As DOX has more polar groups, it showed slower release.	[118]
	Tb/MBG nanospheres	80SiO_2_-15CaO-5P_2_O_5_ doped with 0.5 or 5 mol% Tb_2_O_3_	DOX + Tb^3+^	DOX release was dominated by the quantity of doping tellurium, and pH of the solution.	[120]
	Sm/MBG/alginate	60SiO_2_-36CaO-4P_2_O_5_ doped with 5 or 15 mol% Sm_2_O_3_	DOX + Sm^3+^	The composite showed a sustained drug release. Besides, DOX release was modulated by the samarium quantity and pH environment of the dilution solution.	[151]
	Eu/MBG nanospheres	60%SiO_2_-(36–x)%CaO-x%Eu_2_O_3–_4%P_2_O_5_ (x = 0, 0.5, 1, 2 mol%)	DOX + Eu^3+^	The addition of Eu^3+^ in the synthesis led to changes in pore sizes and surface area, allowing different DOX loading in the MBG. Also, Eu^3+^ increased bioactivity, and the system was cytotoxic against MG-63 osteosarcoma cells.	[119]
	MBG functionalized with amine or isocyanate groups and capped with ATP ^3^ or ε-poly-L-lysine	85%SiO_2–_10%CaO–5%P_2_O_5_ (% mol)	DOX	MBG functionalized with triamine and capped with ATP showed a gate-opening mechanism in a solution containing ALP ^4^, while MBG functionalized with isocyanate and capped with ε-poly-L-lysine was sensitive to pronase. Those MBG capped with ATP were bioactive only after the gate-opening mechanism.	[121]
	Sol-gel-derived bioactive glass nanoparticles functionalized with NH_3_ and grafted with folic acid (FA).	80SiO_2_-16CaO-4P_2_O_5_ (mol%)	Methotrexate (MTX)	MTX was grafted on FA and sustained release in an aqueous solution. Due to FA grafting, the systems could enter HeLa cells by receptor-mediated endocytosis, but only the system BG-FA-MTX was cytotoxic.	[123]
	Fe_2_O_3_/MBG nanocomposite	80SiO-15CaO-5P_2_O_5_ (mol%)	Mitomycin C + magnetic hyperthermia	Mitomycin C release kinetics was dependent on the pH of the release media, being faster delivered at lower pH. Mitomycin C showed toxicity in MG-63 cells.	[124]
	Mg-MBG	51SiO_2_-18CaO-20Na_2_O-4P_2_O_5_-7MgO (mol%)	Mitomycin C	Mitomycin C release kinetics was dependent on the pH of the release media, being faster delivered at lower pH. Mitomycin C showed toxicity in MG-63 cells.	[152]
Kinase inhibitor	MBG	51SiO_2_·20CaO·20Na_2_O·5K_2_O·4P_2_O_5_	Imatinib	Imatinib release kinetics was dependent on the pH of the release media, being faster delivered at lower pH. Imatinib showed toxicity in MG-63 cells.	[125]
Bisphosphonates	MBG nanospheres	80SiO_2_-15CaO-5P_2_O_5_ (mol%)	Alendronate	The drug-delivery system showed cytotoxicity to MG-63 cells, besides promoting in vitro anti-bone absorption response by killing the osteoclast model (RAW 264.7).	[129]
	Melt-derived 13-93 bioactive glass	53SiO_2_-6Na2O-20CaO-12K_2_O-5MgO-4P_2_O_5_ (wt.%)	Zoledronic Acid	The system led to the favorable remodeling of the tubular bone structure in bone fracture regeneration in in vivo models using Sprague-Dawley rats.	[130]
	Melt-derived 45S5 Bioactive glass	45SiO_2_-24.5Na2O-24.5CaO-6P_2_O_5_ (wt.%)	Alendronate	In vivo tests in Sprague-Dawley rats showed bone regeneration, but no anti-osteoclast activity.	[131]
	Pluronic F127 hydrogel/Ho-doped 58S Bioactive glass	58SiO_2_-33CaO-P_2_O_5_ (wt.%)	Zoledronic Acid	Part of the zoledronic acid was encapsulated in the hydrogel (free-ZA), and another part was bonded to the glass (bonded-ZA). The free-ZA was responsible for promoting selective cytotoxicity in MG-63 osteosarcoma cells, but not in MC3T3 pre-osteoblast cells.	[134]
Bioactive compounds and therapeutic ions	Ferrimagnetic bioactive glass grafted with gallic acid	24.7SiO_2_-13.5CaO-13.5Na_2_O-3.3P_2_O_5_-31Fe_2_O_3_-14FeO (wt.%)	Gallic acid + magnetic hyperthermia	Gallic acid showed promoted the formation of ROS species that could be used to cause oxidative stress in cancer cells.	[135]
	Sol-gel-derived Bioactive glass nanoparticle	80SiO_2_-16CaO-4P_2_O_5_ (mol%)	miRNA	The bioactive glass nanoparticles showed higher gene transfection than commercial transfection reagents, such as polyethyleneimine (PEI 25KD) and lipofectamine 3000.	[136]
	MBG nanoparticle	70SiO_2_-30CaO (mol%)	miRNA + DOX	MBG nanoparticles were uptaken by HeLa cells and showed cytotoxicity in transfected cells.	[137]
	45S5 Bioactive glass	45SiO_2_-24.5Na_2_O-24.5CaO-6P_2_O_5_ (wt.%)	n/a	Glasses were able to disrupt the cell membrane of giant cell tumors of the bone and cause necrotic death.	[140]
	MBG nanospheres	80SiO_2_-15CaO-5P_2_O_5_ and 70SiO_2_-25CaO-5P_2_O_5_ (mol%)	Ca^2+^	Ca^2+^ ions activate transient receptor potential channels and calcium-sensing receptors on hepatocellular carcinoma cells (HepG2), signaling cell death by the calpain-1Bcl-2caspase-3 signaling pathway without affecting healthy cells.	[141]
	Zn-MBG	86SiO_2_-14ZnO	Zn^2+^	Zn2+ release was enhanced in the acid microenvironment and caused cytotoxicity in breast cancer cells (MDA-MB-231 and MCF-7 (ER+).	[142]
	Melt—derived bioactive glass	0.51SiO_2_–0.29Na_2_O–(0.20-X)ZnO–XV_2_O_5_, 0 ≤ X ≤ 0.09	Zn^2+^ + V^5+^	The glass showed selective apoptosis of liver cancer cells, which was addressed to vanadium rather than zinc.	[143]
	Sol-gel-derived 58S bioactive glass	58SIO_2_-33CaO-9P_2_O_5_ doped with 1-5 wt.% of Ho_2_O_3_	Ho^3+^ + brachytherapy	Ho-containing bioactive glasses display selective cytotoxicity on human osteosarcoma MG63 cell lineages while favoring high biocompatibility in pre-osteoblast-like cells (MC3T3-E1).	[7,144]
	(Se, Te)-MBG	80SiO_2_-15CaO-5P_2_O_5_ doped with 5 mol% of Se_2_O_4_ or Te_2_O_4_ replacing SiO_2_.	Se^4+^ or Te^4+^	Caused oxidative stress, which was more pronounced in MG-63 cells than in MC3T3.	[153,154,155]

^1^ MBG = mesoporous bioactive glass (all the MBG were synthesized by the sol-gel method), ^2^ PCL = polycaprolactone, ^3^ ATP = adenosine triphosphate, ^4^ ALP = alkaline phosphatase.

## Data Availability

No new data were created in this study. Data sharing is not applicable to this article.
